# Starburst amacrine cells amplify optogenetic visual restoration through gap junctions

**DOI:** 10.1016/j.omtm.2023.05.011

**Published:** 2023-05-12

**Authors:** Yusaku Katada, Hiromitsu Kunimi, Naho Serizawa, Deokho Lee, Kenta Kobayashi, Kazuno Negishi, Hideyuki Okano, Kenji F. Tanaka, Kazuo Tsubota, Toshihide Kurihara

**Affiliations:** 1 Laboratory of Photobiology, Keio University School of Medicine, Shinanomachi, Shinjuku-ku, Tokyo 160-8582, Japan; 2 Department of Ophthalmology, Keio University School of Medicine, Shinanomachi, Shinjuku-ku, Tokyo 160-8582, Japan; 3 Department of Nutritional Sciences, Toyo University, Kita-ku, Tokyo 115-8650, Japan; 4 Section of Viral Vector Development, Center for Genetic Analysis of Behavior, National Institute for Physiological Sciences, National Institutes of Natural Sciences, Okazaki, Aichi 444-8585, Japan; 5 Department of Physiology, Keio University School of Medicine, Shinanomachi, Shinjuku-ku, Tokyo 160-8582, Japan; 6 Division of Brain Sciences, Institute for Advanced Medical Research, Keio University School of Medicine, Shinanomachi, Shinjuku-ku, Tokyo 160-8582, Japan; 7 Tsubota Laboratory, Inc, Shinjuku-ku, Tokyo 160-0016, Japan

**Keywords:** retina, gene therapy, optogenetics, visual restoration, starburst amacrine cells

## Abstract

Ectopic induction of optogenetic actuators, such as channelrhodopsin, is a promising approach to restoring vision in the degenerating retina. However, the cell type-specific response of ectopic photoreception has not been well understood. There are limits to obtaining efficient gene expression in a specifically targeted cell population by a transgenic approach. In the present study, we established a murine model with high efficiency of gene induction to retinal ganglion cells (RGCs) and amacrine cells using an improved tetracycline transactivator-operator bipartite system (KENGE-tet system). To investigate the cell type-specific visual restorative effect, we expressed the channelrhodopsin gene into RGCs and amacrine cells using the KENGE-tet system. As a result, enhancement in the visual restorative effect was observed to RGCs and starburst amacrine cells. In conclusion, a photoresponse from amacrine cells may enhance the maintained response of RGCs and further increase or improve the visual restorative effect.

## Graphical abstract

**Figure:**
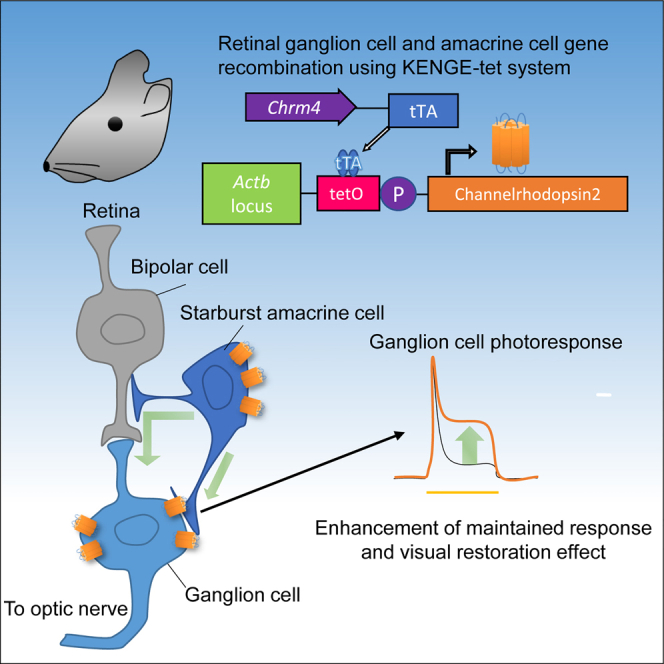

## Teaser

Ectopic induction of optogenetic actuators is a promising approach to restoring vision in the degenerating retina. This study uses the KENGE-tet system for efficient gene induction in retinal ganglion and amacrine cells, revealing that starburst amacrine cells amplify optogenetic visual restoration through gap junctions.

## Introduction

Inherited retinal degeneration is one of the major causes of vision loss. More than 2 million people worldwide are blind because of this disease,^1^ and there is still almost no effective treatment. Previous studies have reported visual restoration effects in animal models by various molecules such as optogenetic actuators.^2^^,^^3^^,^^4^^,^^5^^,^^6^^,^^7^^,^^8^^,^^9^ In addition, clinical trials have also started using channelrhodopsin 2 (RST-001, ClinicalTrials.gov Identifier: NCT01648452) and Chrimson R (GS-030, ClinicalTrials.gov Identifier: NCT03326336), with gene transduction into retinal ganglion cells (RGCs) by intravitreal injection of recombinant adeno-associated virus (rAAV). Although the visual reconstruction effects by optogenetic gene transfer (such as channelrhodopsin-2 into RGCs^2^ and ON-bipolar cells^3^^,^^4^^,^^5^^,^^6^^,^^8^) have been shown, interactions between the other types of cells in the retinal neural circuits in optogenetic visual restoration have not been well understood yet. Channelrhodopsin 2 conductance was reported to be 50–250 fS,^10^ indicating the need for sufficient gene expression to control the membrane potential. In this study, we employed a tetracycline-controllable gene expression system (tet system)^11^ in which the amount of gene expression has been much improved (tetracycline transactivator-operator bipartite [KENGE-tet] system).^12^ Furthermore, we established sufficient gene induction in RGCs and amacrine cells. To investigate cell type-specific visual restorative effects, channelrhodopsin2 (E123T/T159C) was ectopically expressed into those cells by the KENGE-tet system. As a result, we revealed that amacrine cells may play an essential role in retinal neuronal circuits via gap junctions to enhance the optogenetic visual restorative effect.

## Results

### Gene induction of RGCs and starburst amacrine cells in M4-YC and RGCs in 5B-YC

**Figure 1:**
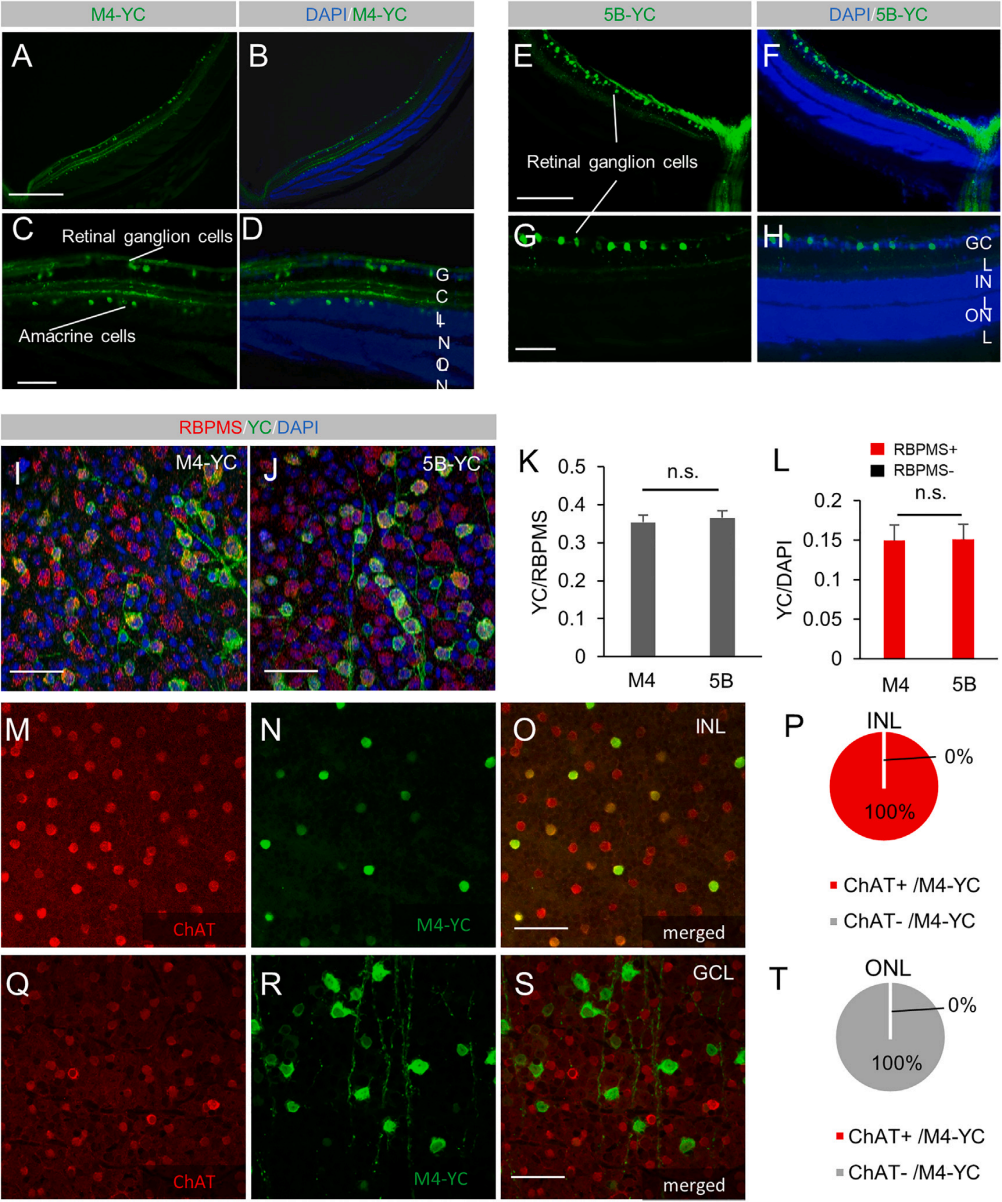
Gene induction of RGCs and SACs in M4-Figure 1YC and RGCs in 5B-YC. In the M4-YC mouse retina, we identified the expression of YC (green) in RGCs and amacrine cells within sections (A–D) In the 5B-YC mouse retina, we identified the expression of YC (green) in RGCs in sections (E–H). Co-expression of the RGC marker RBPMS in flat-mounted retinas of M4-YC(I) and 5B-YC(J). Percentage of YC-positive cells in RBPMS-positive (K) or DAPI-positive cells (L) and RBPMS-positive cells in YC-positive cells (L) in both lines from confocal flat-mounted GCL (n = 3 retinas each). Regions were chosen in each quadrant, and we obtained RBPMS, DAPI-positive, YC-positive, and co-labeled cells. Co-expression of the SAC marker ChAT in flat-mounted retinas of M4-YC in INL (M–O) and GCL (Q–S). Percentage of ChAT-positive cells in YC-positive cells in M4-YC mice from INL (P) and GCL (T) (n = 3 retinas each). Error bars represent the SEMs. INL, inner nuclear layer; ONL, outer nuclear layer. Scale bar, 50 μm in (E); 100 μm in (B–D), (H), (I), (K), (M), (Q), and (R); 400 μm in (N); and 500 μm in (F), (G), (O), and (P).

We used two different mouse lines that express the gene encoding the tetracycline transactivation (tTA) protein under the control of a cell type-specific promoter, muscarinic acetylcholine receptor 4 (*Chrm4*)^13^ or 5-hydroxytryptamine (5-HT [serotonin]) 5B receptor (*Htr5b*)^12^ control region: *Chrm4*-tTA or *Htr5b*-tTA. These mice were further crossed with another transgenic mouse line containing the yellow Cameleon-Nano 50 (YC) fluorescent gene connected into the downstream of the tet operator (tetO) promoter.^14^ The YC gene expression was induced only by the presence of tTA protein in the double transgenic mice (*Chrm4*-tTA:tetO-YC [M4-YC] or *Htr5b* -tTA:tetO-YC [5B-YC]) (Figures S1A and S1B). The expression of YC was observed in the double transgenic mouse retina with a fluorescence microscope. In the M4-YC mouse retina, we identified the expression of YC in RGCs (Figures 1A–1D and S2A–S2E) and amacrine cells (Figures 1A–1D, S1E, and S1F) with *in vivo* fluorescence microscopy and in sections. In the 5B-YC mouse retina, we identified the expression of YC in RGCs (Figures 1E–1H) and the corneal stromal layer (Figures S2G–S2M) with *in vivo* fluorescence microscopy and in sections. In both lines, no expression was found in displaced amacrine cells; only RGCs were labeled in the ganglion cell layer, and the percentage of YC-positive cells in RGC marker RBPMS^15^-positive cells was 35.4% (n = 5) in M4-YC and 36.5% (n = 5) in 5B-YC from RGCs, which were equivalent (p = 0.68) (Figures 1I–1L, 1O–1R, and S2M–S2Q). All of the amacrine cell expression in the M4 line was choline acetyltransferase (ChAT) staining-positive and consisted of starburst amacrine cells (SACs), and 31.1 ± 2.1% (n = 3) of ChAT-positive cells were YC-positive (Figures 1M–1T and S3A–S3L).

As a visual restoration model, the *Chrm4*-tTA and *Htr5b*-tTA lines were crossed with tetO-channelrhodopsin2 (E123T/T159C) (tetO-ChR2) mice (Figure S1C).^16^ *Chrm4*-tTA:tetO-ChR2 (M4-ChR2) drove ChR2 expression in both RGCs and amacrine cells and *Htr5b* -tTA:tetO-ChR2 (5B-ChR2) only in RGCs in the retina. In the retinas of these double transgenic mice, photoreceptor degeneration was induced by intraperitoneal injection of N-methyl-N-nitrosourea (MNU).^17^ MNU is an alkylating agent that causes DNA methylation in the O6 position of guanine, resulting in apoptosis, and it has been widely used to induce a pharmacological animal model of retinitis pigmentosa (RP). Two weeks after 75 mg/kg MNU administration into the mice at the age of 8 weeks, the outer nuclear layer containing photoreceptors was largely absent (Figures S4A and S4B), and the light-evoked response from photoreceptors was not detected by electroretinography (ERG) (Figure S4C).

TUNEL assay was performed to determine whether there was any neurotoxicity-induced cell death in the retina caused by Channel rhodopsin expression; however, we could not observe any cell death in the retina (Figure S4D).

### M4-ChR2 mouse shows higher visual restorative effect

**Figure 2:**
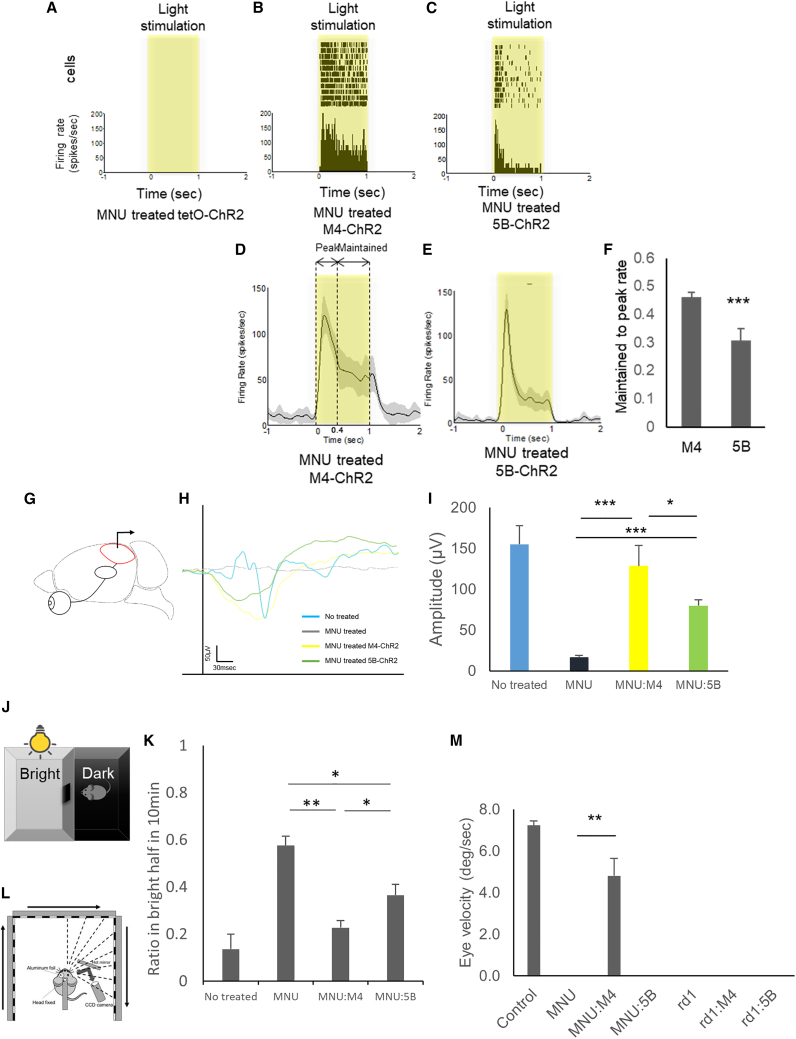
M4-ChR2 mouse shows a higher visual restoration effect (A–C) Raster plots and peri-stimulus time histogram (PSTH) of light stimulation from single RGCs of MNU-treated tetO-ChR, M4-ChR2, and 5B-ChR2 mice. Light intensity was 13.6 log photons/cm^2^/s, and the duration was 1.0 s. (D, E) The averaged rate histogram after filtering with the Gaussian function from M4-ChR2 and 5B-ChR2 mice. At least 10 trials were conducted for each cell. The gray areas around the averaged traces represent the SEM. (F) Maintained-to-peak ratio of the spiking responses recorded. The maintained time frame is 0.4–1.0 s from light stimulation as shown in H (n = 7 retinas, 164 cells in MNU-treated M4-ChR2 mice, and n = 4 retinas, 127 cells in MNU-treated 5B-ChR2 mice). Error bars represent SEMs. ∗∗∗p < 0.001. Student’s 2-tailed t test. (G) Schematic image of VEP measurement. (H) Representative VEP traces from MNU-injected and control mice. (I) The average amplitude of the VEPs in the control tetO-ChR mice (n = 4), MNU-treated tetO-ChR mice (n = 8), M4-ChR2 mice (n = 14), and 5B-ChR2 mice (n = 12) at 10 weeks of age. It was stimulated with a light stimulus intensity of 100-ms pulses of white LED 4,000 cds/m^2^. Signals were low-pass filtered at 300 Hz and averaged over the 60 trials. (J) Schematic image of the LDT testing. Mice were tested in a 30 × 45 × 30-cm box containing equally sized bright (200 lux at ground level) and dark chambers connected by a 5 × 5-cm opening, across which the mice could move freely. Visible mice feel uneasy in bright places, so staying time in the bright half gets shorter. (K) Percent time in the bright half at 10 min of control (tetO-ChR2 mice) (n = 4), MNU-injected tetO-ChR2 mice (n = 8), MNU-injected M4-ChR2 mice (n = 15), and MNU-injected 5B-ChR2 mice (n = 8) measured from LDT testing. (L) Schematic view of the OKR system. The images of the right or left eyes are captured by a CCD camera placed on the same side. During measurement, the contralateral eyes are covered with aluminum foil. Visual stimulation is presented on three LCD monitors around the mouse, the head of which is fixed in the middle. (M) The average eye velocities of the control tetO-ChR2 mice (n = 3), MNU-injected tetO-ChR2 mice (n = 3), M4-ChR2M4-ChR2 mice (n = 9), 5B-ChR2 mice (n = 5), *rd1*;tetO-ChR2 mice (n = 8), *rd1*;M4-ChR2M4-ChR2 mice (n = 10), and *rd1*;5B-ChR2 mice (n = 5) measured from the OKR system at 10 weeks of age. All error bars represent the SEMs. ∗p < 0.05, ∗∗p < 0.01, ∗∗∗p < 0.001. One-way ANOVA testing.

To evaluate the function of the channelrhodopsin ectopically induced in the transgenic mouse retinas, we performed a multi-electrode array (MEA) recording, which can record extracellular potentials of RGCs (Figure S4E). As a result of photoreceptor degeneration induced with MNU treatment, the control retina without channel-rhodopsin expression (tetO-ChR2) showed no response from RGCs detected (Figure 2A). In contrast, the M4-ChR2 (Figure 2B) and 5B-ChR2 (Figure 2C) retinas showed obvious light-induced responses. After filtering with the Gaussian function (Figures 2D and 2E), the maintained response after the peak was significantly greater in M4-ChR2 mice than in 5B-ChR2 mice (Figure 2F), which indicates that the light-evoked ON response in RGCs could be modified through the response from amacrine cells.

To investigate whether light reception on the retina is transmitted to the visual cortex, we then examined visual evoked potentials (VEPs) from the visual cortex (Figure 2G). The output from RGCs is sent through the axons of RGCs (the optic nerve) to the lateral geniculate nucleus (LGN) of the thalamus, which is a region of the mesencephalon, from the LGN to the primary visual cortex (V1) in the occipital lobe of the cerebral cortex. VEPs were not detected in the control tetO-ChR2 mice with MNU treatment (Figures 2H and 2I). In contrast, VEPs were observed in both M4-ChR2 and 5B-ChR2 mice treated with MNU (Figures 2H and 2I). In response to the light stimulus at 4,000 cds/m^2^, the average of the VEP amplitude in MNU-treated M4-ChR2 mice was significantly higher (143.6 μV; n = 12, p = 0.03) than in MNU-treated 5B-ChR2 (79.6 mV; n = 12) and the same level as in the mice without MNU treatment (155 μV; n = 4, p = 0.84). However, the shapes of the waveforms were irregular in both models compared with controls, and their physiological roles are unknown. Thus, there is a limitation in this direct comparison.

To validate the model system, we also examined the MEA, ERG, and VEPs of M4-ChR2 and 5B-ChR2 in the absence of retinal degeneration (no MNU treatment and no mutation). As a result of MEAs, the peak response was significantly decreased in both lines and further decreased with MNU treatment (Figure S4F). A similar trend was observed for the maintained response, whereas it was maintained in the M4 line (Figure S4G). This finding suggests that channelrhodopsin gene induction into healthy RGCs interferes with the physiological retinal light response. In addition, in the control mice, not only the ON response but also the OFF response was confirmed. In contrast, the OFF response was significantly lost in the M4 line (Figure S4I). It is known that SACs in the inner nuclear layer are connected to OFF bipolar cells,^18^ which might be related to this change. As a result of ERG and VEPs, both lines tended to have shorter latencies, especially in photopic examinations (Figures S5E, S5I, S5L, and S5O), likely because of the channelrhodopsin response to strong light^9^ and its photoreception in RGCs resulting in short latency.^2^ Although the amplitude of both ERG and VEP tended to be small in the 5B line (Figure S5C, S5G, S5M, and S5N), these measurements were not significant, and there was no significant change in the shape of the waveforms.

Next, light-dark transition (LDT) testing was performed to investigate whether ectopic expression of channelrhodopsin in the degenerative retinas may lead to behavioral changes according to visual restoration. Rodents tend to stay in dark places according to their visual function; they are nocturnal and feel uneasy in bright environments (Figure 2J), while the visually disturbing MNU treatment (Figure 2K) resulted in almost one-half of the staying time in bright and dark places (ratio in bright half at 10 min was closer to 0.5). In contrast, both the M4-ChR2 and 5B-ChR2 mouse lines with each MNU treatment showed decreased staying time in bright places, indicating that visual restoration was confirmed in these models. Furthermore, M4-ChR2 mice showed significantly higher visual restorative effects (0.23) than 5B-ChR2 mice (0.37; p = 0.043) (Figure 2K).

We also examined the optokinetic response (OKR) to investigate whether light receptivity in the retina restored by ectopic channelrhodopsin expression could lead to central reflex movement output. With the OKR, a rotating striped pattern was displayed to the head-fixed mice to induce eye movements, and the velocity was measured to evaluate the integrity of the subcortical reflex circuitry of the mice (Figure 2L). An OKR was not detected in the mice with MNU administration except for the M4 line (4.80 deg/sec, n = 6) (Figure 2M).

This outcome seems to be caused by a lack of ChR2 expression in SACs, which play a key role in OKR,^19^ in the retinas of 5B-ChR2 mice. We also examined *rd1* mice as a genetic animal model of RP. These mice have a nonsense mutation in the *Pde6b* gene leading, to rapid degeneration of rods and photoreceptors, followed by the loss of cones.^20^^,^^21^ We used blind *rd1* mutation mice at the age of 10–12 weeks for the following experiments. In the cases of *rd1*-mutant mice, no detectable OKR was observed in any combinations with each transgenic line, including M4-ChR2. To investigate the dissociation of OKR between the MNU treatment and *rd1* models, the retinal thickness was compared under the same conditions above. As a result, the total retinal thickness of *rd1* mice (78.3 μm; n = 9) was significantly thinner than that of MNU-treated mice (97.2 μm; n = 10) evaluated on optical coherence tomography (OCT) (Figures S6A and S6B). This outcome was thought to be because the inner retinal layer was thinner of the *rd1* mouse (Figure S6C). Indeed, the inner nuclear layer of the *rd1*, *rd1*;5B-ChR2, and *rd1*;M4-ChR2 mice was significantly thinner than that of the MNU-treated mice (Figure S6D) and YC-positive cells in the inner retinal layer of *rd1*;M4-ChR2 tended to be fewer than those in the inner retinal layer of MNU-treated mice (Figure S6E), suggesting that SACs in the inner layer may be degenerated. This result suggests that residual inner retinal thickness and its durability might be potential limitations of optogenetic gene therapy in inherited retinal disease patients clinically.

### Acetylcholine and gap junctions are involved in the maintained response

**Figure 3:**
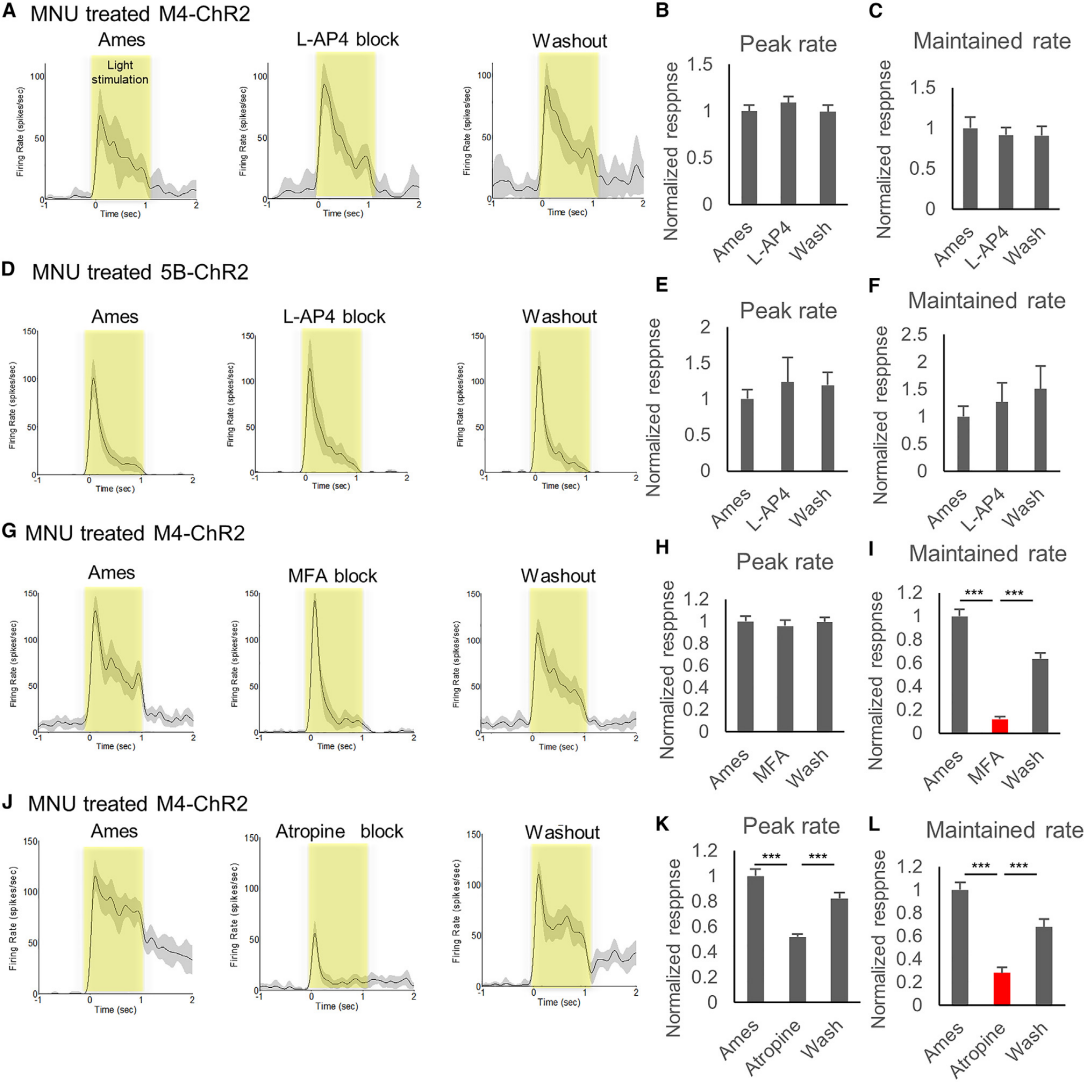
Acetylcholine and gap junctions were involved in the maintained response (A, D, G, J) Mean ± SEM of exemplar cell response firing rate recorded during normal Ames' medium superfusion (left), in synaptic block (middle), and after washout (right). MNU-treated M4-ChR2 mice with L-AP4 block (n = 3 retinas, 47 cells) (A), MNU-treated 5B-ChR2 mice with L-AP4 block (n = 3 retinas, 45 cells) (D), MNU-treated M4-ChR2 mice with MFA block (n = 3 retinas, 85 cells) (G), and MNU-treated M4-ChR2 mice with atropine block (n = 3 retinas, 33 cells) (J). The gray areas around the averaged traces represent the SEM. (B, C, E, F, H, I, K, L) Averaged normalized peak firing rate and maintained rate. The maintained time frame is 0.4–1.0 s from light stimulation. Light intensity was 13.6 log photons/cm^2^/s. All error bars represent the SEM. ∗∗∗p < 0.001. One-way ANOVA and Tukey’s test.

The M4-ChR2 mouse line, which showed the transgene expression in amacrine cells in addition to RGCs, showed a higher maintained response and more effective visual restoration (Figure 2) than the 5B-ChR2 mice. Therefore, we investigated the neurocircuit pathway responsible for the enhanced response caused by amacrine cells using neurotransmitter blockers on the MEA recording. Administration of L-2-amino-4-phosphonobutyric acid (L-AP4), which is an agonist for group III metabotropic glutamate receptors, including mGluR6 working as a blocker of retinal ON-bipolar cells, did not show significant changes in either MNU-treated M4-ChR2 (Figures 3A–3C) or 5B-ChR2 (Figures 3D–3F) retinas, which indicates that the photoresponse was not derived from photoreceptors. In contrast, the administration of mefenamic acid (MFA) or carbenoxolone, an inhibitor of gap junctions, induced a significant decrease (MFA, 0.12, p < 0.001; carbenoxolone, 0.68, p = 0.009) in the maintained response, but not the peak of the response (MFA, 0.96, p = 0.35; carbenoxolone, 1.00, p = 0.78) in the MNU-treated M4-ChR2 retina (Figures 3G–3I and S7A–S7C). This change was recovered after washout and not observed in the 5B-ChR2 retina (Figures S7D–S7F). SACs release GABA and acetylcholine.^22^ Thus, we also examined inhibitors of GABA receptors, bicuculline for GABA-A, CGP 52432 for GABA-B, and (1,2,5,6-tetrahydropyridin-4-yl) methylphosphinic acid (TPMPA) for GABA-C receptors, and cholinergic antagonists, mecamylamine for nicotinic, and atropine for muscarinic acetylcholine receptors. As a result, no significant change was observed with the administration of GABA receptor blockers (Figures S7G–S7O) and mecamylamine (Figures S7P–S7R), but atropine administration showed a similar decrease in the maintained response (0.51, p < 0.001) and the peak response (0.28, p < 0.001) in the MNU-treated M4-ChR2 mice (Figures 3J–3L). In addition, to determine whether this change may occur via bipolar cells, an AMPA receptor antagonist, 6-cyano-7-nitro-quinoxaline-2,3-dione (CNQX), and an NMDA receptor antagonist, D(−)-2-amino-5-phosphonopentanoic acid (D-AP5), were administered. As a result, no statistically significant difference was observed, although there was a tendency toward a decrease in the maintained response (Figures S7S–S7U). SACs form synapses with bipolar cells, RGCs, and other amacrine cells.^22^^,^^23^^,^^24^ It is also known that amacrine cells are directly connected with RGCs through gap junctions,^25^^,^^26^ regulating neural circuits in the retina.^27^ Therefore, these results indicated that SACs enhanced the maintained response of RGCs directly or indirectly through muscarinic acetylcholine and gap junctions.

**Figure 4:**
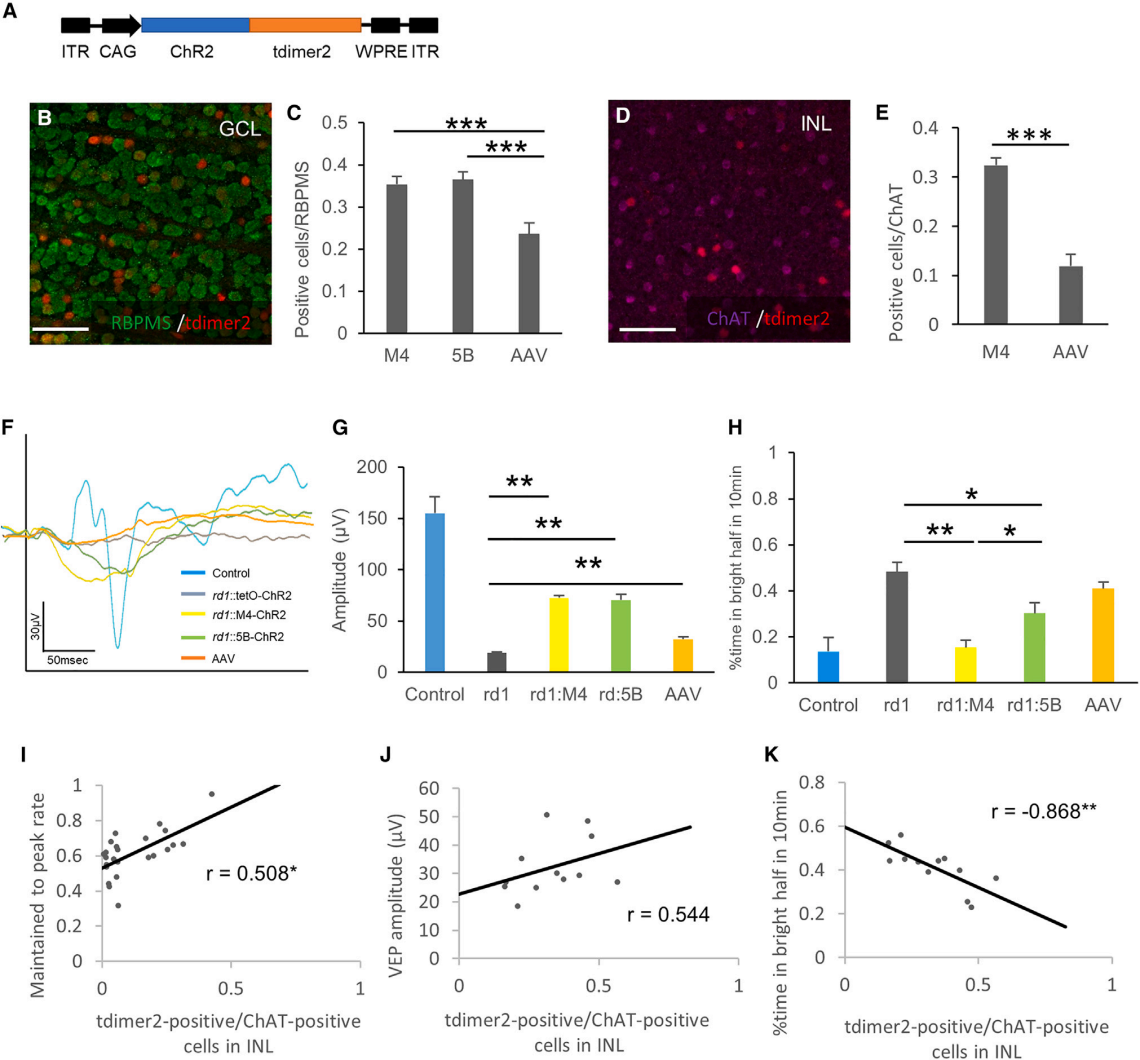
Induction efficiency into SACs tended to affect visual restoration (A) The rAAV2-CAG-ChR2-tdimer2-WPRE expression cassette. ITR, inverted terminal repeat; WPRE, woodchuck hepatitis virus posttranscriptional regulatory element. (B–E) Co-expression of the RGC marker RBPMS (B) and SAC marker ChAT (D) and tdimer2 in flat-mounted retinas of rAAV2-CAG-tdimer2-WPRE treated retinas in *rd1* mice. (C, E) Percentage of YC-positive cells in M4-YC and 5B-YC mice and tdimer2-positive cells in rAAV-treated mice in RBPMS-positive (C) or ChAT-positive cells (E) from confocal flat mounted retina (n = 3 retinas each). Regions were chosen in each quadrant, and we obtained RBPMS, ChAT-positive, YC/tdimer2-positive, and co-labeled cells. (F, G) Average VEP traces (F) and quantification of its amplitudes (G) from control (tetO-ChR2) (n = 3), *rd1*:tetO-ChR2 (n = 6), *rd1*;M4-ChR2 (n = 12), *rd1*;5B-ChR2 (n = 12) and rAAV-treated *rd1*:tetO-ChR2 mice (n = 12) at 10 weeks of age. It was stimulated with 100-ms pulses of white LED 4,000 cds/m^2^ light stimulus intensity. Signals were low-pass filtered at 300 Hz and averaged over the 60 trials. (H) Percent time in bright half at 10 min in control (tetO-ChR2) (n = 3), *rd1*:tetO-ChR2 (n = 6), *rd1*;M4-ChR2 (n = 12), *rd1*;5B-ChR2 (n = 12), and rAAV-treated *rd1*:tetO-ChR2 mice (n = 12) measured from LDT testing. (I–K) Correlation between transfection efficiency into SACs (tdimer2-positive cells/ChAT-positive cells in INL) and maintained to peak rate (I) (n = 24), VEP amplitude (J) (n = 24), and percent time in the bright half in LDT testing (K) (n = 12). All error bars represent the SEMs. INL, inner nuclear layer; GCL, ganglion cell layer. Scale bars, 50 μm in (B), (D), n.s., not significant, ∗p < 0.05, ∗∗p < 0.01, ∗∗∗p < 0.001. unpaired t test (E), Games-Howell test (C, G, H), and Pearson’s correlation coefficient (I–K).

MEA showed a visual restoration effect induced by ectopic ChR2 expression in the degenerative retina (Figure 2). These photoresponses obtained from MEA were all ON responses, as previously reported.^2^^,^^28^^,^^29^^,^^30^ Several slow photoresponses obtained from RGCs were considered to have an intrinsically photosensitive RGC origin,^31^ and they were excluded from the data. The MNU-treated M4-ChR2 retina showed a significantly more pronounced response in the maintained time phase compared with 5B-ChR2. As a result of a neurotransmitter blocking test (Figure 4), the inhibition of muscarinic acetylcholine and gap junctions decreased the sustained response of only M4-ChR2 retinas. The difference between M4-ChR2 and 5B-ChR2 was the presence or absence of ChR2 gene expression in SACs. Therefore, it was suggested that photoresponse from SACs enhanced the maintained response of RGCs, enhancing the visual restoration effect via muscarinic acetylcholine and gap junctions.

### Induction efficiency into SACs tends to affect visual restoration

Finally, to investigate its clinical applicability, the effect of SACs on visual restoration was examined using rAAV2, which was approved for gene therapy.^32^ We injected a rAAV2-CAG-ChR2-tdimer2 intravitreally into *rd1* mice and performed MEA, VEPs, and LDT testing (Figures 4A, S8A and S8B). In addition, we counted the number of transfected RGCs and SACs and analyzed their relevance to visual restorative effects. As a result of immunohistochemical labeling using RBPMS, the infection efficiency to RGCs was 23.6%, which was equivalent to previous reports (21%–23%),^2^^,^^33^^,^^34^ and significantly less than those of our transgenic mice (M4tet 35.4%, p = 0.004; 5Btet 36.5%, p = 0.002) (Figures 4B and 4C). The efficiency of SACs was 11.9%, which was also less than that of transgenic mice (32.4%, p = 0.002) (Figures 4D and 4E). As a result of MEA recordings, the peak response in the rAAV model was equivalent to that of both transgenic lines (Figure S8C). The maintained response and its rate were equal to that of the M4 line (Figures S8D and S8E), perhaps because the input from various cell types other than SACs is also included in the rAAV model. The increased maintained response may be affected by gene transfer to cells other than SACs, but similar characteristics to the M4 line have been obtained. Each average of the VEP amplitude in the *rd1*;M4-ChR2, *rd1*;5B-ChR2, and rAAV-treated *rd1* mice in response to the light stimulus at 4,000 cds/m^2^ was 72.7 μV (n = 10), 70.4 μV (n = 12), and 32.2 μV (n = 12), respectively. These responses were significantly higher than those in *rd1*;tetO-ChR2 mice (19.1 mV; n = 8, p < 0.01 all), but smaller than those in the 8-week-old wild-type C57BL/6J mice (155 μV; n = 4) (Figures 4F and 4G). There was no significant difference between *rd1*;5B-ChR2 and *rd1*; M4-ChR2 mice (p = 0.99), and the VEPs of rAAV-treated *rd1* mice were smaller than those of them (Figures 4F and 4G). Even in the LDT results, the restorative effect of rAAV-treated *rd1* mice tended to be lower than that of transgenic mice (Figure 4H), perhaps because of its infection efficiency or the higher maintained response might not lead directly and simply to better visual restoration. In addition, the correlation between the infection efficiency of SACs in each rAAV-treated *rd1* mouse and the visual restorative effect was investigated. The results showed a significant, positive correlation between the number of transfected SACs in the inner nuclear layer and the maintained to peak ratio in MEA (Figure 4I), a positive correlation between the VEP amplitudes (Figure 4J), and a significant, negative correlation between the time spent in the bright half in LDT testing (Figure 4K). This outcome suggests that the maintained response in MEA is derived from SACs and that, clinically, the inclusion of SACs, rather than only RGCs, which is the primary target of optogenetic therapy, is effective for visual restoration. Although there is a limitation because infection efficiency is a confounding factor, there was no correlation between the number of transfected RGCs in the ganglion cell layer and the maintained to peak ratio in MEA (Figure S8F). The correlation of the number of transfected SACs in the inner nuclear layer with the VEP amplitudes (Figure 4J), and the time spent in the bright half in LDT testing (Figure 4K) was stronger than that with infection efficiency into RGCs (Figures S8G and S8H).

## Discussion

In this study, we established transgenic mouse lines inducing gene induction in specific retinal cells using the tet system (Figure S1). We identified that gene induction occurred in RGCs and SACs under the control of a muscarinic acetylcholine receptor *Chrm4* promoter. In contrast, 5-HT *Htr5b* receptor control region led to induction only in RGCs in the retina (Figure 2). Among acetylcholine receptors expressed by all types of neurons in the retina,^35^^,^^36^^,^^37^^,^^38^^,^^39^^,^^40^ the *Chrm4* muscarinic receptor is expressed in RGCs and amacrine cells.^35^^,^^41^ All of the amacrine cell expressions in the M4 line consisted of type-a SACs (Figures 1M–1T and S3A–S3L). There are two subtypes of SACs, type-a, and type-b (displaced SACs).^42^^,^^43^ Within the scope of our knowledge, we could not find any reports that describe a transgenic line specific to type-a SACs exclusively, without affecting type-b SACs. Approximately 30% of the type-b SACs were expressed in the M4 line, and no noticeable morphological differences were observed between positive and negative SACs. The 5-HT *Htr5b* receptor is expressed in rodents, but not in humans.^44^ Although several types of 5-HT receptors are expressed in the mouse retina,^45^ the retinal distribution of the *Htr5b* receptor has not been previously described to our knowledge. We found that these gene inductions in both mouse retinas are useful for examining the functions of RGCs and SACs.

Accumulating data have shown that the visual restoration strategy induced by optogenetic genes is a promising therapy for degenerative retinal diseases. Most amacrine cells are inhibitory neurons in the vertebrate retina, which have not been regarded much in the elementary visual restoration target. This study showed that SACs increased the maintained response through gap junctions and contributed to enhancing the visual restorative effect. In particular, since the restorative effect of LDT testing and OKR was more significant than that of VEPs, it might contribute to sustained behavior and direction recognition rather than transient response, consistent with the role of the maintained response in RGCs.^46^ Including the results using rAAV, in the current situation, viral delivery in primates is limited,^28^ and gene transfer involving amacrine cells, for example, using ubiquitous promoters, might be more effective in visual restoration than limiting RGCs in the clinical setting.

In conclusion, this study suggests that the optogenetic photoresponse from SACs enhances the maintained response, which further enhances the visual restoring effect. These results may provide the basis for advanced visual restoration in the future.

## Materials and methods

### Key resources

Key resources are presented in Table 1.

**Table 1:** Key resource table

Reagent or resource	Source	Identifier
**Chemicals, peptides, and recombinant proteins**		

N-Nitroso-N-methylurea	Toronto Research Chemicals	M325815
Domitor (medetomidine hydrochloride)	Zenoac	
Antisedan (atipamezole)	Zenoac	
Midazolam Sandoz	Sandoz	
Vetorphal (butorphanol)	Meiji Seika Pharma	
Ames’ medium	Sigma	A1420
L-AP4	Abcam	ab120002
Meclofenamic acid sodium salt	Sigma	M4531
TPMPA	TOCRIS	1040
Bicuculline	Wako	026–16131
CGP 52432 hydrochloride	Sigma	SML0593
Mecamylamine	TOCRIS	2843/10
CNQX	Abcam	ab120044
D-AP5	Abcam	ab120003
Carbenoxolone	MERCK	C4790
Atropine	MERCK	A0132
DAPI	Life Technologies	D21490
Anti-RBPMS antibody	Abcam	ab194213
Anti-ChAT antibody	Abcam	ab114p
Anti-VGluT2	Frontier Institute	VGlut2-Rb-Af720
Anti-syntaxin antibody	Abcam	ab3265
Anti-tyrosine hydroxylase antibody	Abcam	ab112
Anti-Prox1 antibody	AngioBio	11-002P
pAAV CAG ChR2 E123T T159C 2A tDimer	Addgene	#85399

**Experimental models: Organisms and strains**		

Mouse: C3H/HeJJcl	CLEA Japan	
Mouse: tetO-YCnano50	RIKEN BRC	RBRC09550
Mouse: tetO-ChR2 (E123T/T159C)	RIKEN BRC	RBRC05843
Mouse: *Chrm4*-tTA	RIKEN BRC	RBRC09551
Mouse: *Htr5b*-tTA	RIKEN BRC	RBRC05445

**Oligonucleotides**		

Genotyping primer: tet-forward: CATGAAGCAGCACGACTTCTT		
Genotyping primer: tet-reverse: TTCTTACTTGTACAGCTCGTCCA		
Genotyping primer: M4-forward: AAGCACCAAGTTCTCTCCCGTCTT		
Genotyping primer: M4-reverse: CGGAGTTGATCACCTTGGACTTGT		
Genotyping primer: 5B-forward: GCTCCAGGAAACCACAATGCCTTT		
Genotyping primer: 5B-reverse: CGGAGTTGATCACCTTGGACTTGT		
Genotyping primer: *rd1*-forward: AAGCTAGCTGCAGTAACGCCATTT		
Genotyping primer: wild-forward: ACCTGCATGTGAACCCAGTATTCTATC		
Genotyping primer: *rd1* wild common-reverse: CTACAGCCCCTCTCCAAGGTTTATAG		

### Animals

Transgenic mice used for the experiments and their genotyping protocols were generated as previously reported.^12^ Mice homozygous for the retinal degeneration alleles Pde6b^*rdl*^ (C3H/HeJJcl, *rd1*) and WT C57BL/6J were obtained from CLEA Japan, Inc. Animals were maintained under 12-h light:12-h dark conditions. For animals bred in-house, littermates of the same sex were randomized to experimental groups. Mice used for the experiments were heterozygous for the tTA and tetO genes and homozygous for the *rd1* gene. All of the animal experiments were conducted under protocols approved by the Institutional Animal Care and Use Committee of Keio University School of Medicine.

tetO-YC (RBRC09550), tetO-ChR2 (RBRC05843), *Chrm4*-tTA (RBRC09551), and *Htr5b*-tTA (RBRC05445) are available from RIKEN Bioresource Center in Japan.

### Study approval

All of the animal experiments were conducted under protocols approved by the Institutional Animal Care and Use Committee of Keio University School of Medicine (#2808).

### Immunohistochemistry

The protocol for immunohistochemistry as previously described.^47^ The retinas were incubated in PBS with 1% Triton X-100 and 0.5% Tween 20 for 1 h at room temperature and in 4% BSA for 1 h at room temperature and then incubated overnight at 4°C with primary antibodies: RBPMS (1:500, Abcam), ChAT (1:100, Abcam), VGlut2 (1:100, Frontier Institute), syntaxin (1:100, Abcam), tyrosine hydroxylase (1:100, Abcam) and Prox1 (1:100, AngioBio) in blocking buffer. Secondary anti-rabbit, mouse, and goat IgG, conjugated with Alexa TM488, TM594, and 633, respectively (1:1,000; Molecular Probes), were applied for 1 h at room temperature. The retinas then were flat mounted, and the sections were mounted on slide glass. The TUNEL assay was performed based on our previous reports.^48^^,^^49^

### Vector production and purification

pAAV-CAG–ChR2(E123T/T159C)-tdimer2-WPRE (Addgene, ) was used for the current study. Type 2 serotypes of rAAV vectors were prepared using the AAV Helper Free Packaging System (Cell Biolabs). The serotypes were produced in HEK293 cells using a helper virus-free system and were purified using two CsCl_2_ density gradients and titrated by quantitative polymerase chain reaction. Final preparations were dialyzed against PBS and stored at −80°C.

### Virus injection

The mice were anesthetized with a combination of midazolam, medetomidine, and butorphanol tartrate at doses of 4 mg/kg, 0.75 mg/kg, and 5 mg/kg of body weight and placed on a heating pad that maintained their body temperatures at 35°C–36°C throughout the experiments. An aperture was made subsequent to the limbus through the sclera with a 30G disposable needle, and a 33G unbeveled blunt tip needle on a Hamilton syringe was introduced through the scleral opening into the vitreous space for intravitreal injections and introduced through the scleral opening along the scleral interior wall into the subretinal space for subretinal injections. Each eye received 1 μL vehicle (PBS) or vector at a titer of 2.0 × 10^11^ vg/mL.

### MEA recordings

All of the MEA procedures were performed under a dim red light. The mice were anesthetized and euthanized by quick cervical dislocation. After enucleation, the retina was dissected at room temperature in Ames’ medium bubbled with 95% O_2_/5% CO_2_ (A 1420; Sigma-Aldrich). The separated retina was placed on a cellulose membrane, and RGCs were directed to the electrode and was gently contacted against MEA (MEA2100-Systems; Multi-Channel Systems) under suction pressure. During the experiment, the retinas were continuously perfused with Ames’ medium bubbling at 34°C at a rate of 1–2 mL/min. Recorded signals were collected, amplified, and digitized using MC Rack software (Multi-Channel Systems). Retinas were perfused for 30 min in darkness before recording responses. A 470-nm blue LED light was used at 1.0 × 10^16^ photons·cm^−2^·s^−1^ stimulus. L-(+)-2-amino-4-phosphonobutyric acid (L-AP4, ab120002; Abcam), TPMPA (Cat. No. 1040; Tocris Bio-Science), meclofenamic acid sodium salt (MFA, M4531; Sigma-Aldrich), atropine (A0132; MERCK), carbenoxolone (C4790; MERCK), Bicuculline (026–16131; Wako), CGP52432 (SML0593; Sigma), and Mecamylamine (2843/10, TOCRIS) were newly diluted to 20 μM, 20 μM, 100 μM, 20 μM, 100 μM, 10μM, 20 μM, and 20 μM, respectively. Stimulation was presented for 1 s at 60-s intervals. Signals were filtered between 200 Hz (low cutoff) and 20 kHz (high cutoff). A threshold of 40 μV was used to detect action potentials, and action potentials from individual neurons were determined via a standard expectation-maximization algorithm using Offline Sorter software (Plexon). The results were plotted using NeuroExplorer software (Nex Technologies). Maintained-to-peak amplitude ratio was calculated by dividing the maintained response amplitude in maintaining time frame (0.4–1.0 s after light stimulation) by the peak amplitude (this ratio quantifies the sustenance of the response).

### ERG analyses

Scotopic ERG was recorded according to our previous report.^47^ Animals were dark adapted for 12 h and prepared under dim red illumination. The pupils were dilated with a mixed solution of 0.5% tropicamide and 0.5% phenylephrine (Mydrin-P; Santen). Then, the mice were anesthetized with a combination of midazolam, medetomidine, and butorphanol tartrate at doses of 4 mg/kg, 0.75 mg/kg, and 5 mg/kg of body weight, respectively, and were placed on a heating pad that maintained their body temperature at 35°C–36°C throughout the experiments. The ground electrode was a subcutaneous needle in the tail, and the reference electrode was placed subcutaneously between the eyes. The active contact lens electrodes (Mayo) were placed on the corneas. Recordings were performed with a PuREC acquisition system (Mayo). Responses were filtered through a bandpass filter ranging from 0.3 to 500 Hz to yield a- and b-waves. White LED light stimulations of 10.0 log cd-s/m^2^ were delivered via a Hemisphere LS-100 Stimulator (Mayo).

### VEP analyses

The measuring electrodes for VEP analyses were placed more than 1 week before the measurement. The mice were anesthetized with a combination of midazolam, medetomidine, and butorphanol tartrate at doses of 4 mg/kg, 0.75 mg/kg, and 5 mg/kg of body weight, respectively. The animals were placed in a stereotaxic holder. A stainless steel screw (M1.0 × 6.0 mm) inserted through the skull into both visual cortex (1.5 mm laterally to the midline, 1.5 mm anterior to the lambda), penetrating the cortex to approximately 1 mm, served as a measuring electrode.

At the time of measurement, mice were anesthetized again with the same doses. Visual stimuli were generated by a white LED placed 3 cm from the eye. It was stimulated with 100-ms pulses of white LED 4,000 cds/m^2^ light stimulus intensity. Signals were acquired and analyzed with a PuREC acquisition system (Mayo). Signals were low-pass filtered at 300 Hz and averaged over the 60 trials.

### OKR recording system

Protocols for eye movement recording and visual stimulation were previously described.^50^^,^^51^ Eye movements were recorded from both eyes of each animal separately. During the recording, the contralateral eye was covered by aluminum foil. The head of the mouse was fixed to an experimental steel board by the head-mounted stick for the LIM lens frames. The reflected images through a hot mirror (43957-J, Edmund) were recorded using an infrared CCD camera (BS-GV200, Libraly Inc.). The images of the eye movements were processed and analyzed using software (Move-tr/2D, Libraly Inc.). The sampling rate of the image was 200 Hz. The center of the pupil was detected by the software. We calculated the speed of the eye movements on two-dimensional images and converted them to angular speeds using the axial length of each eye. The spatial frequency was set as 0.125 cycles/degree, and the temporal frequency of the visual stimulus was 1.5 Hz. The motion onset delay (MOD) was set as 333 ms. Continuing the MOD, sinusoidal grating started to move clockwise in 5 s. The intervals of the visual stimulus were 60 s. Eye movements were recorded three times for each experiment to exclude shaking images caused by excessive body movements. The average velocities of the eye movements were calculated in the slow speed phase of their nystagmus.

### LDT recording

Mice were tested in a 30 × 45 × 30-cm box containing equally sized light and dark chambers connected by a 5 × 5-cm opening via which mice could move freely. The bright half of the box was illuminated from above by a white fluorescent light with an intensity of 200 lux measured at the floor level. The animals were placed in the bright half, and movement was recorded (HD Pro Webcam C920, Logitech). A trial lasted 10 min, and then the testing apparatus was dismantled and cleaned with 70% ethanol. Videos were analyzed using ANY-maze tracking software and were validated by comparison with manual analysis. Time spent in the bright half was recorded.

### Preparation of whole-mount samples and cryosections of retinas

Enucleated eyes were fixed for 20 min in 4% paraformaldehyde (PFA) in PBS and then dissected as previously described.^52^ The obtained tissues were post-fixed overnight in 4% PFA and stored in methanol at −20°C. Cryosections of retinas (12 mm) were prepared as previously described^53^ after the eyeballs were immersed overnight in 4% PFA. Retinal sections were observed using a confocal microscope (LSM710; Carl Zeiss).

### OCT imaging

The thickness of the retina was analyzed by an SD-OCT system (Envisu R4310; Leica) tuned for mice. The imaging protocol entailed a 3 × 3-mm perimeter square scan sequence producing a single *en face* image of the retina through a 50° field of view from the mouse lens, following mydriasis. The *en face* image consisted of 100 B-scan tomograms, with each B-scan consisting of 1,000 A-scans. The retinal thickness of 150 μm from the optic disc of each quadrant was measured.

### Quantification and statistical analyses

All of the results are expressed as the mean ± SEM. The averaged variables were compared using the Student’s 2-tailed t test and the Kruskal Wallis one-way ANOVA test. p-values of less than 0.05 were considered statistically significant.

## Data and software availability

Raw MEA spike data were sorted offline to identify single units using Offline Sorter software (version 4.4.0) (Plexon). Spike-sorted data were analyzed with NeuroExplorer 5 software (version 5.115) (Nex Technologies).

## Supplemental information

Document S1. Figures S1–S8

Document S2. Article plus supplemental information

## References

[1] Sahel J.A., Marazova K., Audo I. (2014). Clinical characteristics and current therapies for inherited retinal degenerations. Cold Spring Harb. Perspect. Med..

[2] Bi A., Cui J., Ma Y.P., Olshevskaya E., Pu M., Dizhoor A.M., Pan Z.H. (2006). Ectopic expression of a microbial-type rhodopsin restores visual responses in mice with photoreceptor degeneration. Neuron.

[3] Doroudchi M.M., Greenberg K.P., Liu J., Silka K.A., Boyden E.S., Lockridge J.A., Arman A.C., Janani R., Boye S.L., Boye S.E. (2011). Virally delivered channelrhodopsin-2 safely and effectively restores visual function in multiple mouse models of blindness. Mol. Ther..

[4] Lagali P.S., Balya D., Awatramani G.B., Münch T.A., Kim D.S., Busskamp V., Cepko C.L., Roska B. (2008). Light-activated channels targeted to ON bipolar cells restore visual function in retinal degeneration. Nat. Neurosci..

[5] Cronin T., Vandenberghe L.H., Hantz P., Juttner J., Reimann A., Kacsó A.E., Huckfeldt R.M., Busskamp V., Kohler H., Lagali P.S. (2014). Efficient transduction and optogenetic stimulation of retinal bipolar cells by a synthetic adeno-associated virus capsid and promoter. EMBO Mol. Med..

[6] Macé E., Caplette R., Marre O., Sengupta A., Chaffiol A., Barbe P., Desrosiers M., Bamberg E., Sahel J.A., Picaud S. (2014;23(August). Targeting channelrhodopsin-2 to ON-bipolar cells with vitreally administered AAV restores ON and OFF visual responses in blind mice. Mol. Ther..

[7] Busskamp V., Duebel J., Balya D., Fradot M., Viney T.J., Siegert S., Groner A.C., Cabuy E., Forster V., Seeliger M. (2010). Genetic reactivation of cone photoreceptors restores visual responses in retinitis pigmentosa. Science.

[8] Van Wyk M., Pielecka-Fortuna J., Löwel S., Kleinlogel S. (2015). Restoring the on switch in blind retinas: opto-mGluR6, a next-generation, cell- tailored optogenetic tool. PLoS Biol..

[9] Pienaar A., Bedford R., Davis K., Bishop P.N. (2015). Restoration of vision with ectopic expression of human rod opsin. Curr. Biol..

[10] Lin J.Y., Lin M.Z., Steinbach P., Tsien R.Y. (2009). Characterization of engineered channelrhodopsin variants with improved properties and kinetics. Biophys. J..

[11] Gossen M., Freundlieb S., Bender G., Müller G., Hillen W., Bujard H. (1995). Transcriptional activation by tetracyclines in mammalian cells. Science.

[12] Tanaka K.F., Matsui K., Sasaki T., Sano H., Sugio S., Fan K., Hen R., Nakai J., Yanagawa Y., Hasuwa H. (2012). Expanding the repertoire of optogenetically targeted cells with an enhanced gene expression system. Cell Rep..

[13] Takata N., Sugiura Y., Yoshida K., Koizumi M., Hiroshi N., Honda K., Yano R., Komaki Y., Matsui K., Suematsu M. (2018). Optogenetic astrocyte activation evokes BOLD fMRI response with oxygen consumption without neuronal activity modulation. Glia.

[14] Kanemaru K., Sekiya H., Xu M., Satoh K., Kitajima N., Yoshida K., Okubo Y., Sasaki T., Moritoh S., Hasuwa H. (2014). In Vivo visualization of subtle, transient, and local activity of astrocytes using an ultrasensitive Ca2+ indicator. Cell Rep..

[15] Rodriguez A.R., de Sevilla Müller L.P., Brecha N.C. (2014). The RNA binding protein RBPMS is a selective marker of ganglion cells in the mammalian retina. J. Comp. Neurol..

[16] Tsunematsu T., Ueno T., Tabuchi S., Inutsuka A., Tanaka K.F., Hasuwa H., Kilduff T.S., Terao A., Yamanaka A. (2014). Optogenetic manipulation of activity and temporally controlled cell-specific ablation reveal a role for MCH neurons in sleep/wake regulation. J. Neurosci..

[17] Tsubura A., Yoshizawa K., Kuwata M., Uehara N. (2010). Animal models for retinitis pigmentosa induced by MNU ; disease progression , mechanisms and therapeutic trials Histology and. Published online.

[18] Vaney D.I., Sivyer B., Taylor W.R. (2012). Direction selectivity in the retina: symmetry and asymmetry in structure and function. Nat. Rev. Neurosci..

[19] Yoshida K., Watanabe D., Ishikane H., Tachibana M., Pastan I., Nakanishi S. (2001). A key role of starburst amacrine cells in originating retinal directional selectivity and optokinetic eye movement. Neuron.

[20] Carter-Dawson L.D., LaVail M.M., Sidman R.L. (1978). Differential effect of the rd mutation on rods and cones in the mouse retina. Invest. Ophthalmol. Vis. Sci..

[21] LaVail M.M., Sidman R.L., Association for Research in Vision and Ophthalmology.LD (1977).

[22] Taylor W.R., Smith R.G. (2012). The role of starburst amacrine cells in visual signal processing. Vis. Neurosci..

[23] Yamada E.S., Dmitrieva N., Keyser K.T., Lindstrom J.M., Hersh L.B., Marshak D.W. (2003). Synaptic connections of starburst amacrine cells and localization of acetylcholine receptors in primate retinas. J. Comp. Neurol..

[24] Yonehara K., Balint K., Noda M., Nagel G., Bamberg E., Roska B. (2011). Spatially asymmetric reorganization of inhibition establishes a motion-sensitive circuit. Nature.

[25] Marc R.E., Sigulinsky C.L., Pfeiffer R.L., Emrich D., Anderson J.R., Jones B.W. (2018). Heterocellular coupling between amacrine cells and ganglion cells. Front. Neural Circ..

[26] Völgyi B., Chheda S., Bloomfield S.A. (2009). Tracer coupling patterns of the ganglion cell subtypes in the mouse retina. J. Comp. Neurol..

[27] Bloomfield S.A., Völgyi B. (2009). The diverse functional roles and regulation of neuronal gap junctions in the retina. Nat. Rev. Neurosci..

[28] Sengupta A., Chaffiol A., Macé E., Caplette R., Forster V., Marre O., Lin J.Y., Desrosiers M., Sahel J.A., Picaud S. (2016). Red-shifted channelrhodopsin stimulation restores light responses in blind mice , macaque retina , and human retina. EMBO Mol Med.

[29] Pan Z.H., Ganjawala T.H., Lu Q., Ivanova E., Zhang Z. (2014). ChR2 mutants at L132 and T159 with improved operational light sensitivity for vision restoration. PLoS One.

[30] Wu C., Ivanova E., Zhang Y., Pan Z.H. (2013). rAAV-mediated subcellular targeting of optogenetic tools in retinal ganglion cells in vivo. PLoS One.

[31] Mure L.S., Hatori M., Zhu Q., Demas J., Kim I.M., Nayak S.K., Panda S. (2016). Melanopsin-encoded response properties of intrinsically photosensitive retinal ganglion cells. Neuron.

[32] Keeler A.M., Flotte T.R. (2019). Recombinant adeno-associated virus gene therapy in light of luxturna (and zolgensma and glybera): where are we, and how did we get here?. Annu. Rev. Virol..

[33] Chaffiol A., Caplette R., Jaillard C., Brazhnikova E., Desrosiers M., Dubus E., Duhamel L., Macé E., Marre O., Benoit P. (2017). A new promoter allows optogenetic vision restoration with enhanced sensitivity in macaque retina. Mol. Ther..

[34] Lin B., Koizumi A., Tanaka N., Panda S., Masland R.H. (2008). Restoration of visual function in retinal degeneration mice by ectopic expression of melanopsin. Proc. Natl. Acad. Sci. USA.

[35] Strang C.E., Renna J.M., Amthor F.R., Keyser K.T. (2010). Muscarinic acetylcholine receptor localization and activation effects on ganglion response properties. Invest. Ophthalmol. Vis. Sci..

[36] Keyser K.T., MacNeil M.A., Dmitrieva N., Wang F., Masland R.H., Lindstrom J.M. (2000). Amacrine, ganglion, and displaced amacrine cells in the rabbit retina express nicotinic acetylcholine receptors. Vis. Neurosci..

[37] Dmitrieva N.A., Strang C.E., Keyser K.T. (2007). Expression of alpha 7 nicotinic acetylcholine receptors by bipolar, amacrine, and ganglion cells of the rabbit retina. J. Histochem. Cytochem..

[38] Liu J., McGlinn A.M., Fernandes A., Milam A.H., Strang C.E., Andison M.E., Lindstrom J.M., Keyser K.T., Stone R.A. (2009). Nicotinic acetylcholine receptor subunits in rhesus monkey retina. Invest. Ophthalmol. Vis. Sci..

[39] Blute T.A., Strang C., Keyser K.T., Eldred W.D. (2003). Activation of the cGMP/nitric oxide signal transduction system by nicotine in the retina. Vis. Neurosci..

[40] Cimini B.A., Strang C.E., Wotring V.E., Keyser K.T., Eldred W.D. (2008). Role of acetylcholine in nitric oxide production in the salamander retina. J. Comp. Neurol..

[41] Strang C.E., Long Y., Gavrikov K.E., Amthor F.R., Keyser K.T. (2015). Nicotinic and muscarinic acetylcholine receptors shape ganglion cell response properties. J. Neurophysiol..

[42] Famiglietti E.V. (1983). Starburst” amacrine cells and cholinergic neurons: mirror-symmetric on and off amacrine cells of rabbit retina. Brain Res..

[43] Famiglietti E.V. (1985). Starburst amacrine cells: morphological constancy and systematic variation in the anisotropic field of rabbit retinal neurons. J. Neurosci..

[44] Grailhe R., Grabtree G.W., Hen R. (2001). Human 5-HT5 receptors: the 5-HT5A receptor is functional but the 5-HT5B receptor was lost during mammalian evolution. Eur. J. Pharmacol..

[45] Chen Y., Palczewska G., Mustafi D., Golczak M., Dong Z., Sawada O., Maeda T., Maeda A., Palczewski K. (2013). Systems pharmacology identifies drug targets for Stargardt disease–associated retinal degeneration. J. Clin. Invest..

[46] Ikeda H., Wright M.J. (1972). Receptive field organization of ‘sustained’ and ‘transient’ retinal ganglion cells which subserve different functional roles. J. Physiol..

[47] Katada Y., Kobayashi K., Tsubota K., Kurihara T. (2019). Evaluation of AAV-DJ vector for retinal gene therapy. PeerJ.

[48] Lee D., Nakai A., Miwa Y., Tomita Y., Serizawa N., Katada Y., Hatanaka Y., Tsubota K., Negishi K., Kurihara T. (2021). Retinal dysfunction induced in a mouse model of unilateral common carotid artery occlusion. BioMed Res. Int..

[49] Kunimi H., Lee D., Ibuki M., Katada Y., Negishi K., Tsubota K., Kurihara T. (2021). Inhibition of the HIF-1α/BNIP3 pathway has a retinal neuroprotective effect. Faseb. J..

[50] Tabata H., Shimizu N., Wada Y., Miura K., Kawano K. (2010). Initiation of the optokinetic response (OKR) in mice. J. Vis..

[51] Jiang X., Kurihara T., Kunimi H., Miyauchi M., Ikeda S.I., Mori K., Tsubota K., Torii H., Tsubota K. (2018). A highly efficient murine model of experimental myopia. Sci. Rep..

[52] Kubota Y., Hirashima M., Kishi K., Stewart C.L., Suda T. (2008). Leukemia inhibitory factor regulates microvessel density by modulating oxygen-dependent VEGF expression in mice. J. Clin. Invest..

[53] Kurihara T., Ozawa Y., Shinoda K., Nagai N., Inoue M., Oike Y., Tsubota K., Ishida S., Okano H. (2006). Neuroprotective effects of angiotensin II type 1 receptor (AT1R) blocker, telmisartan, via modulating AT1R and AT2R signaling in retinal inflammation. Invest. Ophthalmol. Vis. Sci..

